# Wood–Water Relations Affected by Anhydride and Formaldehyde Modification of Wood

**DOI:** 10.1021/acsomega.2c04974

**Published:** 2022-11-08

**Authors:** Muhammad Awais, Michael Altgen, Tiina Belt, Venla Teräväinen, Mikko Mäkelä, Daniela Altgen, Martin Nopens, Lauri Rautkari

**Affiliations:** † Department of Bioproducts and Biosystems, Aalto University, P.O. Box 16300, Aalto, 00076Espoo, Finland; ‡ Department of Biology, Institute of Wood Science, Universität Hamburg, Leuschnerstraße 91c, 21031Hamburg, Germany; § Natural Resources Institute Finland (Luke), Viikinkaari 9, 00790Helsinki, Finland; ∥ VTT Technical Research Centre of Finland Limited, P.O. Box 1000, VTT, FI-02044Espoo, Finland; ⊥ Federal Research Institute for Rural Areas, Forestry and Fisheries, Institute of Wood Research, Johann Heinrich Von Thünen Institute, Leuschnerstrasse 91, 21031Hamburg, Germany

## Abstract

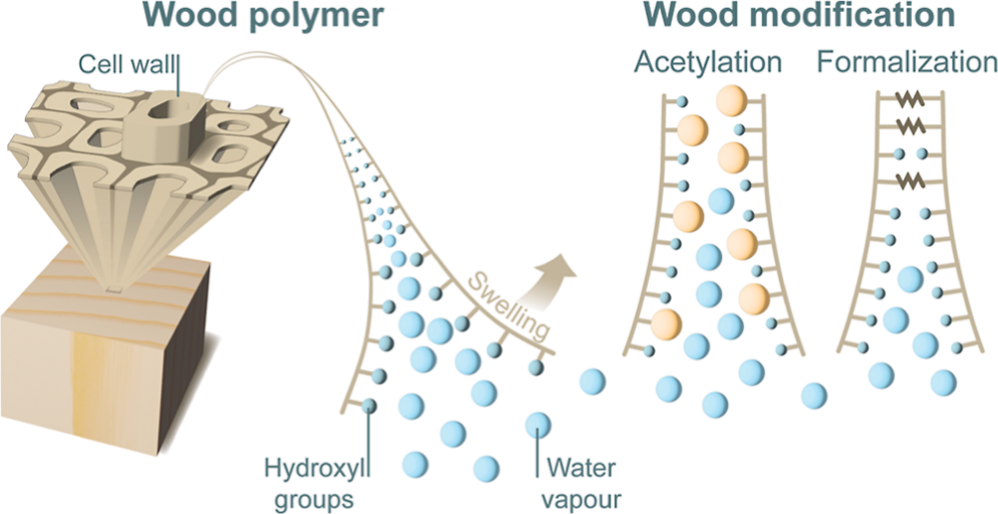

The moisture uptake of wood is influenced by accessible hydroxyl groups acting as sorption sites and the water-available cell wall space. To what extent do these mechanisms control the moisture uptake in wood needs to be addressed. For this purpose, we modified sorption site density and cell wall space by wood treatments with acetic anhydride or formaldehyde and investigated their effects on moisture uptake. Chemical changes at the cell wall level caused by the treatments were first determined by confocal Raman imaging. Following this, the deuterium exchange method was used to gravimetrically measure the hydroxyl accessibility, while the moisture uptake and the consequent swelling of the wood were determined by dynamic measurements of mass and dimensions within the hygroscopic range. The results showed that the effectiveness in reducing the moisture content of untreated wood across the hygroscopic range differed between the anhydride- and formaldehyde-modified wood. We also observed a poor correlation of accessible hydroxyl concentration in formaldehyde-modified wood with weight percentage gain and water uptake. Moreover, the dynamic mass and dimension analysis indicated that the reduction in swelling in formalized wood was affected by an unidentified mechanism in addition to reduced moisture content.

## 1 Introduction

Wood and other lignocellulose materials absorb water either in contact with liquid water or from water vapor in the surrounding atmosphere. Water enters the cell wall through mass flow or diffusion of the water vapor into cell lumens and diffuses from there into the cell wall and causes anisotropic swelling from the dry state.^1^ All the polymeric constituents in the cell wall, such as hemicellulose, cellulose, and lignin, contain hydroxyl groups and therefore influence the wood water relations.^2−5^ The equilibrium moisture content (EMC) of wood is strongly affected by the hydroxyl groups and their accessibility within the hygroscopic range (0–95% RH).^6,7^ A large number of hydroxyl groups are confined within the compact formation of aggregated cellulose microfibrils and are inaccessible to water molecules under normal conditions.^8,9^ However, it has also been reported that the water uptake varies independently from the accessible hydroxyl groups, and a poor correlation was found between the EMC and the OH accessibility.^10−12^ Other factors such as the spatial availability of the cell wall for water molecules and cell wall cross-linking also influenced the water absorption in wood.^13,14^ There is a complex relationship between the absorption or desorption of water molecules, the presence of accessible sorption sites, and the available cell wall space for water. These mechanisms are not very well understood but must be related to the cell wall structure of the wood. In this work, we studied these mechanisms by modifying the wood with acetic anhydride and formaldehyde and by determining the consequent changes in wood–water relations.

Chemical wood modification forms additional covalent bonds within the cell wall and reduces the wood’s affinity for moisture.^15^ Acetylation involves the substitution of hydroxyl groups within the cell wall polymers with acetyl groups, resulting in a single chemical bond per OH group. This reduces the available OH groups for hydrogen bonding with water molecules. Additionally, the added acetyl groups occupy cell wall space to cause a permanent cell wall bulking, which reduces the available cell wall space for water.^16−19^ In formalization, formaldehyde diffuses into the cell wall to react with up to two hydroxyl groups and potentially forms cross-links between two adjacent hydroxyl groups.^18,20^ A catalyst is required to catalyze the reaction of formaldehyde with wood.^16^ In contrast to acetylation, which reduces the available cell wall space for water by cell wall bulking, cross-links formed by formaldehyde treatments may also restrict the swelling of the cell wall.^21^

In this study, we compared the effects of wood modification with acetic anhydride and formaldehyde on wood–water interactions. Chemical changes caused by the treatments were analyzed with confocal Raman imaging and Fourier-transform infrared (FTIR) spectroscopy. The consequent changes in wood–water relations were investigated by hydrogen–deuterium exchange, sorption isotherm measurements, and the determination of simultaneous changes in mass and dimensions within the hygroscopic range. The results revealed significant differences between the two modification methods, which may help in better understanding the mechanisms involved in the water interactions of native wood.

## 2 Methods

### 2.1 Sample Preparation and Modification

The wood samples with dimensions of 20 × 20 × 10 mm^3^ (*R* × *T* × *L*) were cut from the sapwood regions of scots pine (*Pinus sylvestris*) blocks. An experimental design included five design locations to define the trend in chemical modification. Five replicates were prepared at each location, and two additional design locations were designated for reference and catalyst-treated samples. A separate set of wood samples with dimensions of ca. 30 × 30 × 1.5 mm (*R* × *T* × *L*) was prepared to measure the simultaneous mass and dimensional changes during water vapor sorption. All woodblocks were Soxhlet-extracted with acetone for 6 h and oven-dried at 103 °C for 24 h. The initial dry mass of the samples was measured after oven drying at 103 °C for ca. 24 h and cooling in a desiccator over silica gel.

Acetylation was performed as described in our recent study.^22^ In short, the extracted samples were impregnated with neat acetic anhydride in vacuum and at room temperature for 2 h. The acetylation reaction was conducted at 120 °C in a reaction flask under flux. Different time steps were included within the range of 0, 10, 20, 50, 100, and 360 min. The reaction flask was placed in an ice bath to terminate the reaction. The samples were rinsed with fresh acetone, and unreacted acetic anhydride was removed by Soxhlet extraction before the samples were dried in the oven at 103 °C for 24 h. The final dry mass was measured, and the weight percentage gain (WPG) was calculated according to Eq 1
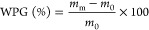
1where *m*_m_ is the dry mass (g) of modified samples, and *m*_0_ represents the initial dry mass (g) of the wood blocks.

For formalization, the extracted samples were impregnated with an aqueous solution of Lewis acid (1.5% ZnCl_2_) for 2 h at 0.04 MPa. The samples were dried in the oven for 24 h at 103 °C, and the dry mass was determined. A set of five samples was separated from the Lewis acid-treated samples and used as a reference for the formalization treatment. The formalization reaction was performed in the vapor phase by placing wood samples above 20 g of paraformaldehyde (CH_2_O)_*n*_ with no direct contact in a desiccator of volume 1000 cm^3^. The desiccator was heated in an oven at 100 °C for 6, 12, 24, 30, or 48 h. The reaction was terminated by removing the samples from the desiccators after cooling for 2 h at room temperature. Soxhlet extraction with acetone was performed again before the final dry mass of the samples was determined after oven-drying. The WPG (%) was calculated as described above.

### 2.2 Infrared Spectroscopy

FTIR spectra of untreated wood, Lewis acid-treated wood, and formalized and acetylated samples were measured using an FTIR spectrometer (Spectrum Two, PerkinElmer, USA). Samples with the highest WPG were selected and milled with a laboratory mill. The spectrometer was equipped with an ATR unit and a diamond crystal. The spectra were measured within the wavenumber range of 1800–800 cm^–1^ at a resolution of 0.25 cm^–1^ and 10 accumulations. Four replicates were measured, and the acquired spectra were baseline-corrected with polynomial fit followed by vector normalization.

### 2.3 Confocal Raman Imaging

Due to the brittleness of the formalized wood blocks, Raman images were acquired from the cross-sectional surfaces of microtome-smoothened wood blocks rather than from thin sections. The images were collected using a Renishaw InVia Qontor confocal Raman microscope equipped with a 785 nm diode laser, a 100× air objective (NA 0.9), and a Centrus 05TJ55 CCD detector behind an 830 lines/mm grating. The images contained 75 lines per image and 75 points per line at a step size of 0.5 μm and were acquired using a 0.5 s integration time. Raman images were area-integrated based on the wave numbers range of 1550–1700, 1640–1680, and 1710–1770 cm^–1^. The selected spectral regions were preprocessed by image despike filter median and baseline corrected with a linear fit. The area was measured with the trapezoidal integration method which approximates the integration by breaking down the area under the curve into finite intervals. After imaging, the average spectra were extracted from the cell wall and cell corner regions of each image. The average spectra were baseline-corrected but not normalized. The image processing was performed by an in-house MATLAB (The MathWorks., Inc.) script.

### 2.4 Deuterium Exchange

The measurements were performed on dried milled powder from each sample group. Samples were placed in the pan of an automated sorption balance (DVS ET, Surface Measurement Systems, UK) under a nitrogen flow (grade 5.0 ≤ 0.5 ppm H_2_O) of 200 sccm. Each sample was first dried by heating to 60 °C for 6 h using the pre-heater followed by cooling to reach 25 °C for 2 h. The dried sample was exposed to deuterium oxide (D_2_O) vapor for 12 h at a target RH of 95%, and the dry mass was determined again as described above. Duplicates of each sample group were measured, and the concentration of accessible hydroxyl groups (OH_acc_, mmol g^–1^) was calculated according to Eq 2
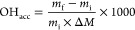
2where *m*_i_ and *m*_f_ are the initial and final dry mass (mg) of the sample, respectively, and Δ*M* is the difference in atomic mass between deuterium and protium (1.006 g mol^–1^).

The concentration of absorbed D_2_O molecules in the deuteration step (3) was calculated by Eq 3
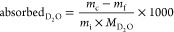
3where *m*_c_ is the mass (mg) of the samples after conditioning to D_2_O vapor, and *M*_D_2_O_ is the molar mass of D_2_O (20.028 g mol^–1^).

The concentration of accessible hydroxyl groups and the concentration of absorbed D_2_O were corrected for the weight added by the modification by multiplication with the correction factor given in Eq 4
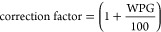
4

In addition to the deuterium exchange measurements, the hydroxyl accessibility in formalized and acetylated wood was also estimated based on the increase in dry sample mass during the treatment and the molecular mass of the functional groups added. The theoretical hydroxyl accessibility of acetylated wood, OH_ac,theo_, was determined using Eq 5
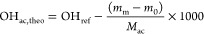
5where OH_ref_ (mmol g^–1^) is the concentration of accessible hydroxyl groups in untreated wood, and *M*_ac_ (g mol^–1^) is the molar mass added by each acetyl group by the reaction of acetic anhydride with one hydroxyl group (42.02 g mol^–1^). The theoretical hydroxyl accessibility of formalized wood, OH_f,theo_, was determined based on Eq 6.
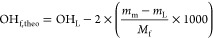
6where OH_L_ (mmol g^–1^) represents the concentration of accessible hydroxyl groups in Lewis acid-treated wood, *m*_L_ (g) is the dry mass after Lewis acid treatment, and *M*_f_ (g mol^–1^) is the molar mass added by the reaction of one formaldehyde molecule with two hydroxyl groups (12.01 g mol^–1^). Note that Eq 6 assumes that each formaldehyde molecule reacts with two hydroxyl groups in wood.

### 2.5 Sorption Isotherms

The samples with the highest WPG (%) from each treatment were milled into a fine powder using a laboratory mill. The sorption isotherms were measured with an automated sorption apparatus (DVS intrinsic, Surface Measurement Systems, UK) that recorded the sample mass during the exposure to nitrogen mixed with water vapor. Approximately 18 mg of wood powder was placed on the sample pan and kept at a constant temperature of 25 °C under a nitrogen flow (grade 5.0; ≤3 ppm H_2_O) of 200 sccm. Absorption and desorption isotherms were measured in 10 relative humidity steps (0, 5, 15, 25, 35, 45, 55, 65, 75, 85, and 95%). Each step was held until the mass change per minute (d*m*/d*t*) was less than 0.0005% min^–1^ over 10 min. The slope in a 10 min window was used to calculate the d*m*/d*t*, using the dry sample mass at the end of the first 0% RH step as reference mass. The moisture content of each sample was calculated using Eq 7
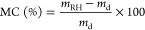
7where *m*_RH_ shows the sample mass (g) after conditioning at specific relative humidity, and *m*_d_ represents the initial dry mass (g) of the modified sample. Moisture content was corrected by the WPG using the correction factor given in Eq 4. In contrast to MC, which relates the mass of absorbed water to the dry sample mass, the corrected moisture content (MC_R_) relates the water mass to the dry wood mass, without the added chemicals.

### 2.6 Simultaneous Mass and Dimensional Changes during Water Vapor Sorption

Water vapor sorption and swelling behavior were determined with a gravimetric sorption system equipped with a high-resolution camera. For the determination of mass and dimensional changes during absorption from 0 to 85% RH, additional wood samples with dimensions of ca. 30 × 30 × 1.5 mm^3^ (*R* × *T* × *L*) were treated as described above. Treatment times of 360 min and 48 h were chosen for acetylation and formalization, respectively. Before the sorption analyses, the samples were soaked in deionized water for ca. 24 h and fixated between two metal meshes to obtain flat cross sections during the subsequent drying at 20 °C and 65% RH. A wood shaper was used to obtain clean radial and tangential surfaces, resulting in final dimensions of ca. 20 × 20 × 1.5 mm^3^ (*R* × *T* × *L*). A 2 mm hole was drilled in the middle of each sample to mount the samples on custom-designed sample holders as described by Nopens et al.^23^ Two unmodified samples, two samples treated with ZnCl_2_, three formalized samples, and three acetylated samples were placed onto the large objects tray of the automated sorption balance (SPSx-1μ-High-Load, ProUmid, Germany). The samples were conditioned at a constant temperature of 20 °C and an RH sequence of 0, 5, 15, 25, 35, 45, 55, 65, 75, and 85%, which was generated by mixing water vapor with dry air. The mass of each sample was determined every 15 min using a balance with a resolution of 1 μg and repeatability of ±5 μg. Simultaneously, an image was taken from the cross section of each sample using a CCD camera (BASLER acA2040-25gc). Each RH step was held until the mass change of each sample was less than 0.01% h^–1^. A moving average in a 1 h window was used as the reference mass to calculate the mass change. After reaching the equilibrium criterion, the RH steps at 0 and 85% were held for additional 5 days.

### 2.7 Image Analysis

**Figure 1:**
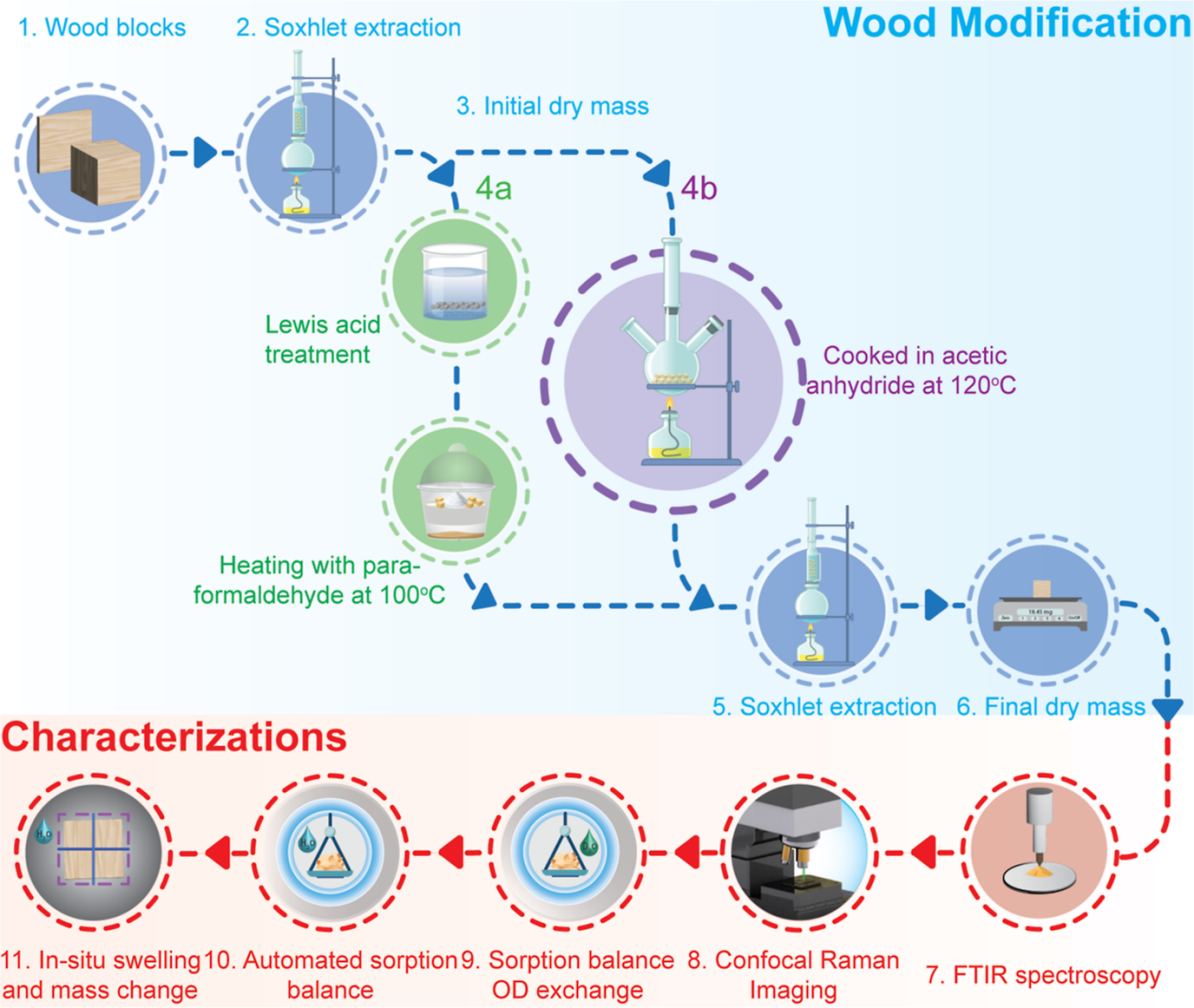
Schematic illustration of the process flow for the chemical modifications and characterizations.

A customized batch macro was generated using Fiji,^24^ an ImageJ distribution.^25^ RGB images were converted into single-color images. Based on threshold images, the cross-sectional areas of the wood samples were calculated by considering the image resolution of ca. 0.016 mm/pixel. The outline of each cross-sectional area was exported as a raster graphic (Figure S1). Images were imported into MATLAB and transformed into pixeled arrays. A linear fit on 1000 data points was applied on each edge, and the distance between two parallel projected lines was calculated in the radial and tangential directions. Dimensional changes (*S*_A_ in %) were determined for each sample and each time point using Eq 8
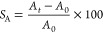
8where *A*_*t*_ is the cross-sectional area (in mm^2^) at the given time point, and *A*_0_ is the cross-sectional area (in mm^2^) at the end of the initial drying step at 0% RH. Dimensional changes in the radial and tangential directions were calculated in the same way, using the corresponding radial and tangential lengths (in mm), respectively. The schematic illustration of the process flow for chemical modifications and characterizations is shown in Figure 1.

## 3 Results and Discussion

### 3.1 Reaction Kinetics and Weight Percentage Gain

**Figure 2:**
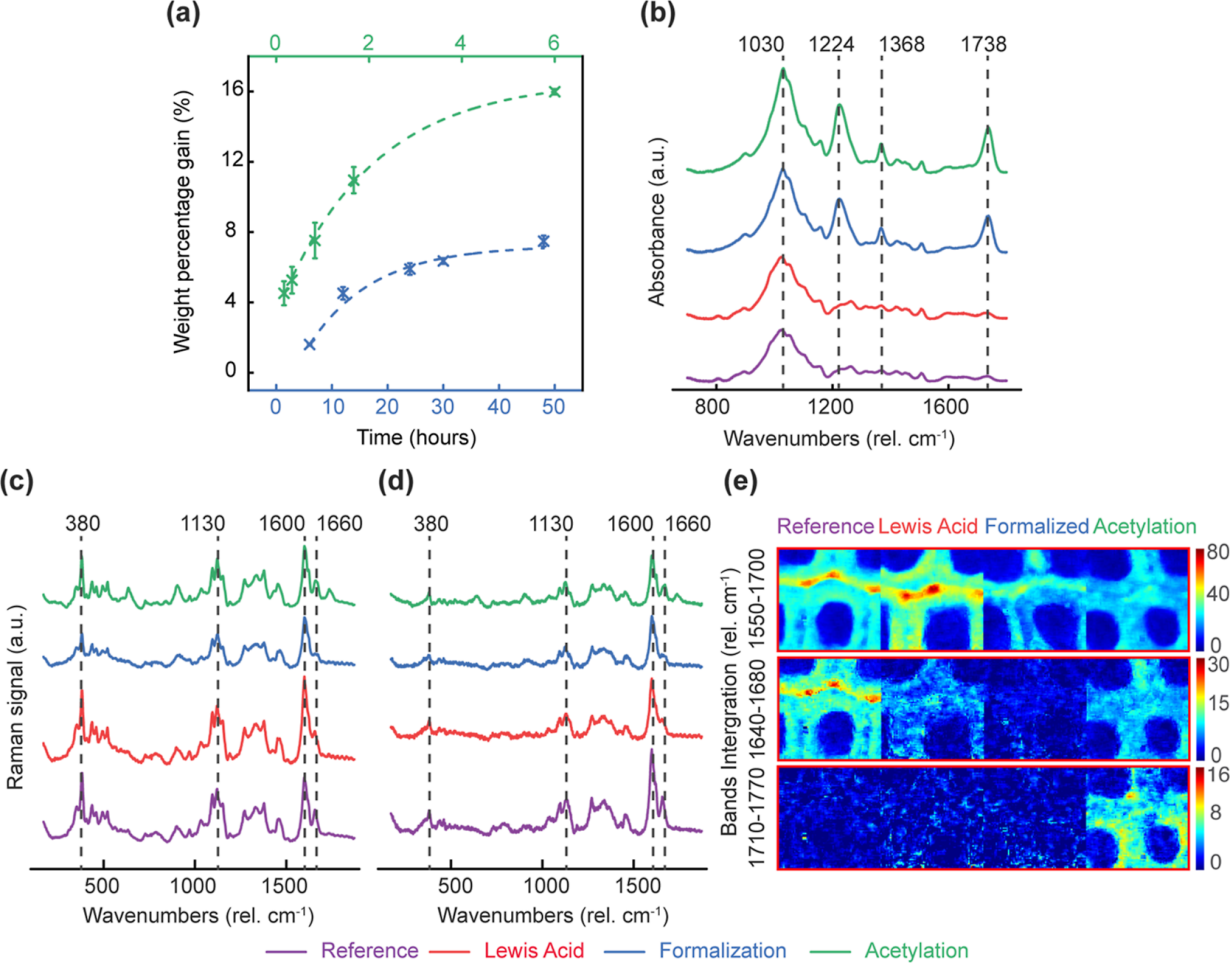
WPG of treated wood, FTIR spectroscopy, and confocal Raman imaging. (a) WPG as a function of time (hours). Please note the different periods for the two treatments. The axis in green shows the time course of acetylation, and the axis in blue is the time course of formalization. The dotted lines show an exponential fit. (b) Example of baseline-corrected FTIR spectra in the wavenumber range 700–1800 cm^–1^. (c) Baseline-corrected average spectra extracted from the cell walls regions of the images in panel (e). (d) Baseline-corrected average spectra extracted from the cell corners regions of the images in panel (e). (e) Raman images based on band integration in the range 1550–1700 rel. cm^–1^ (lignin), 1710–1770 rel. cm^–1^ (acetylation), and 1640–1680 rel. cm^–1^ (Lewis acid treatment/formalization). The overall intensities are different, which is caused by differences in surface quality.

The reaction kinetics of acetylation and formalization modification were measured by determining the WPG for each sample. For acetylation, the woodblocks were soaked in neat acetic anhydride in a reaction flask at a temperature of 120 °C, which resulted in the formation of ester bonds with accessible hydroxyl groups. Wood blocks rapidly achieved 8% WPG within 1 h, followed by a lower reaction rate to 16% WPG after 6 h (Figure 2a). In contrast to acetylation, the formalization reaction was conducted in the vapor phase. Upon heating, the gaseous formaldehyde molecules penetrated the cell walls to form covalent bonds with the hydroxyl groups in wood. One formaldehyde molecule can react with up to two OH groups to form a cross-link between the two reacted groups.^20^ The WPG increased to 4.5% within the first 12 h of exposure before the reaction rate slowed down to yield 6% WPG after 48 h (Figure 2a). Differences in WPG between acetylation and formalization were related not only to different reaction rates of acetic anhydride and formaldehyde with hydroxyl groups but also to the different molecular weights of the functional groups added. At a given WPG, more formaldehyde molecules than acetic anhydride molecules had reacted with the wood because each acetyl group added more mass than one molecule of formaldehyde.

### 3.2 Chemical Changes Caused by the Treatments

FTIR spectroscopy confirmed the chemical changes caused by the treatments within the wave number region of 800–1800 cm^–1^ (Figure 2b). Both acetylation and formalization increased the intensity of carbonyl-derived bands at 1030, 1225, 1371, and 1735 cm^–1^. These bands are commonly observed in native wood. However, the rise in these bands was caused by the treatments because of the formation of ester bonds.^26−28^ There were no noticeable band changes after the Lewis acid treatment.

The average spectra extracted from cell walls and cell corners of confocal Raman images showed that the main spectral change caused by acetylation was the appearance of a band at 1735 cm^–1^, which could be assigned to the C=O stretching of carbonyl groups^29^ (Figure 2c,d). Lewis acid treatment in turn caused a reduction in the intensity of the band at 1660 cm^–1^ while formalization caused the 1660 cm^–1^ band to disappear altogether. The 1660 cm^–1^ band can be assigned to the C=C stretching of the ethenyl moiety of coniferyl alcohol,^30^ and its disappearance could be explained by the polymerization/degradation of monolignols during modification. No other notable spectral changes were detected in the treated samples. The overall intensity differences between the samples had no chemical meaning as they were simply caused by differences in surface quality. The Raman images produced by band integration in the 1550–1700 cm^–1^ region (aromatic lignin stretching vibration at ca. 1600 cm^–1^) showed the distribution of lignin in the samples^31^ and revealed the cell structure in the samples, while the images produced by band integration in the 1640–1680 cm^–1^ (C=C stretching of the ethenyl moiety of coniferyl alcohol at ca. 1660 cm^–1^) and 1710–1770 cm^–1^ (C=O stretching of carbonyl groups at ca. 1735 cm^–1^) regions showed the effects of Lewis acid treatment/formalization and acetylation, respectively (Figure 2e). The images indicated that all three treatments affected both the cell wall and cell corner regions of the samples and that there were no gradients in treatment intensity within the cell walls.

### 3.3 Deuterium Exchange

**Figure 3:**
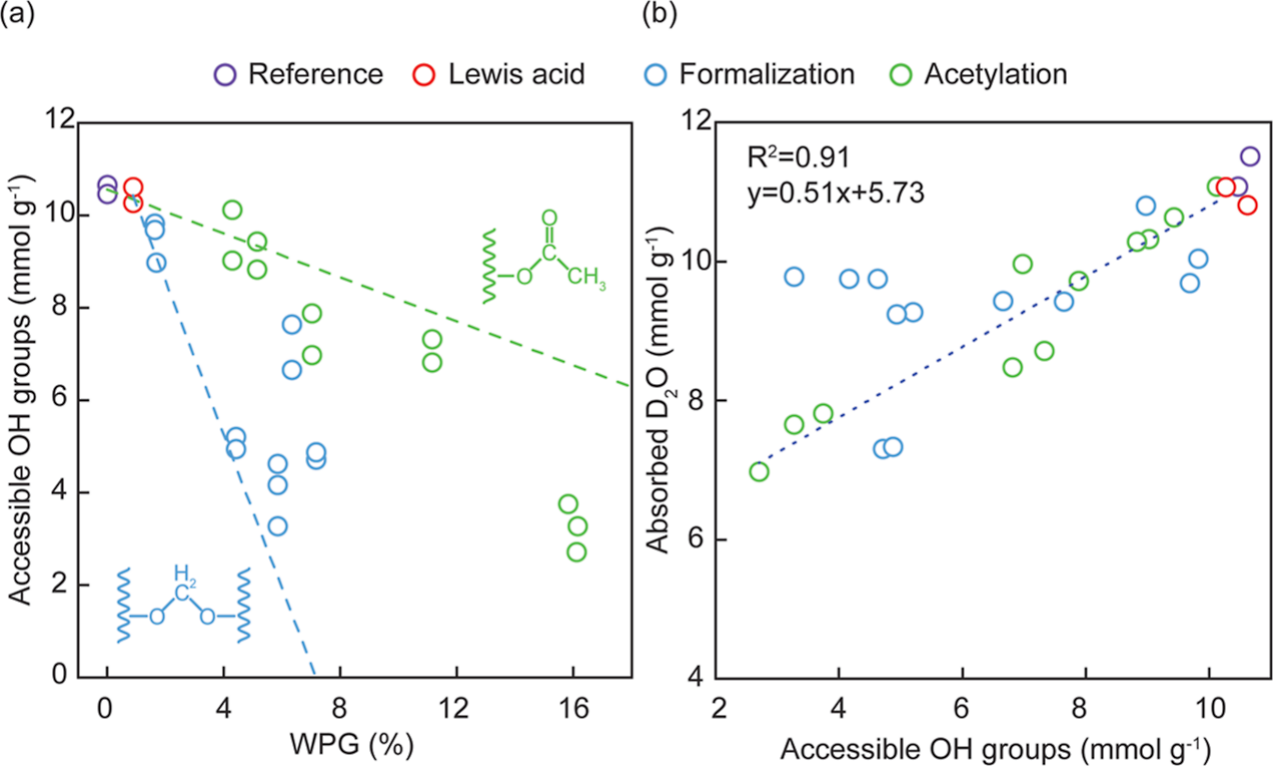
H–D exchange measurements on wood powder. (a) Accessible OH groups (mol g^–1^) as a function of WPG (%). Theoretical estimation was based on the chemical substitution of available hydroxyl groups with acetyl (green) and formaldehyde (blue) molecules. (b) Measured absorbed D_2_O (mmol g^–1^) as a function of accessible OH groups (mmol g^–1^). The dotted lines show a linear fit.

The concentration of OH groups that were deuterated in D_2_O vapor was corrected for the weight added by the treatments using the factor given by Eq 4 and is shown as a function of the WPG in Figure 3. The linear lines in Figure 3a show the theoretical estimation of accessible OH groups considering the chemical substitution of available hydroxyl groups with acetyl and formaldehyde molecules. The measured data points for acetylation decreased with increasing WPG but were mostly below the theoretical concentrations of accessible OH groups. Presumably, this was because steric hindrance caused by the acetyl substitution may have blocked the access to some remaining OH groups.^16^ The theoretical calculations of the accessible OH group concentration in formalized wood assumed that each formaldehyde molecule reacted with two OH groups. However, the measured accessible OH group concentrations mostly exceeded this theoretical concentration. This indicated that some formaldehyde molecules reacted with only one OH group and, hence, did not reduce the number of sorption sites effectively. There was large data scattering in formalized samples, possibly because the ratio of formaldehyde molecules that reacted with one or two OH groups varied between the samples.

The concentration of absorbed D_2_O at 95% target RH was also calculated as a function of accessible OH groups, which showed how the moisture content reduction by the two treatments correlated with the loss in sorption sites (Figure 3b). Both concentrations were corrected for the weight added by the treatments using the factor given by Eq 4. Absorbed D_2_O increased linearly with the increase in accessible OH groups (*R*^2^ = 0.9) with a slope of ca. 0.5, which may suggest that the removal of one absorbed water molecule requires the substitution of two accessible OH groups. However, an earlier study showed that the reduction in wood moisture content by treatments with carboxylic acid anhydrides was not determined by the concentration of accessible OH groups, but by the water-available cell wall space.^14,32^ The data points of formalized wood were scattered above and below the same regression line. The lack of a clear correlation between absorbed water and accessible OH groups suggested that other factors controlled the moisture content of formalized wood. Lewis acid treatment had no effect on the concentration of absorbed D_2_O molecules or on the accessible OH groups when compared to untreated wood.

### 3.4 Sorption Isotherms

**Figure 4:**
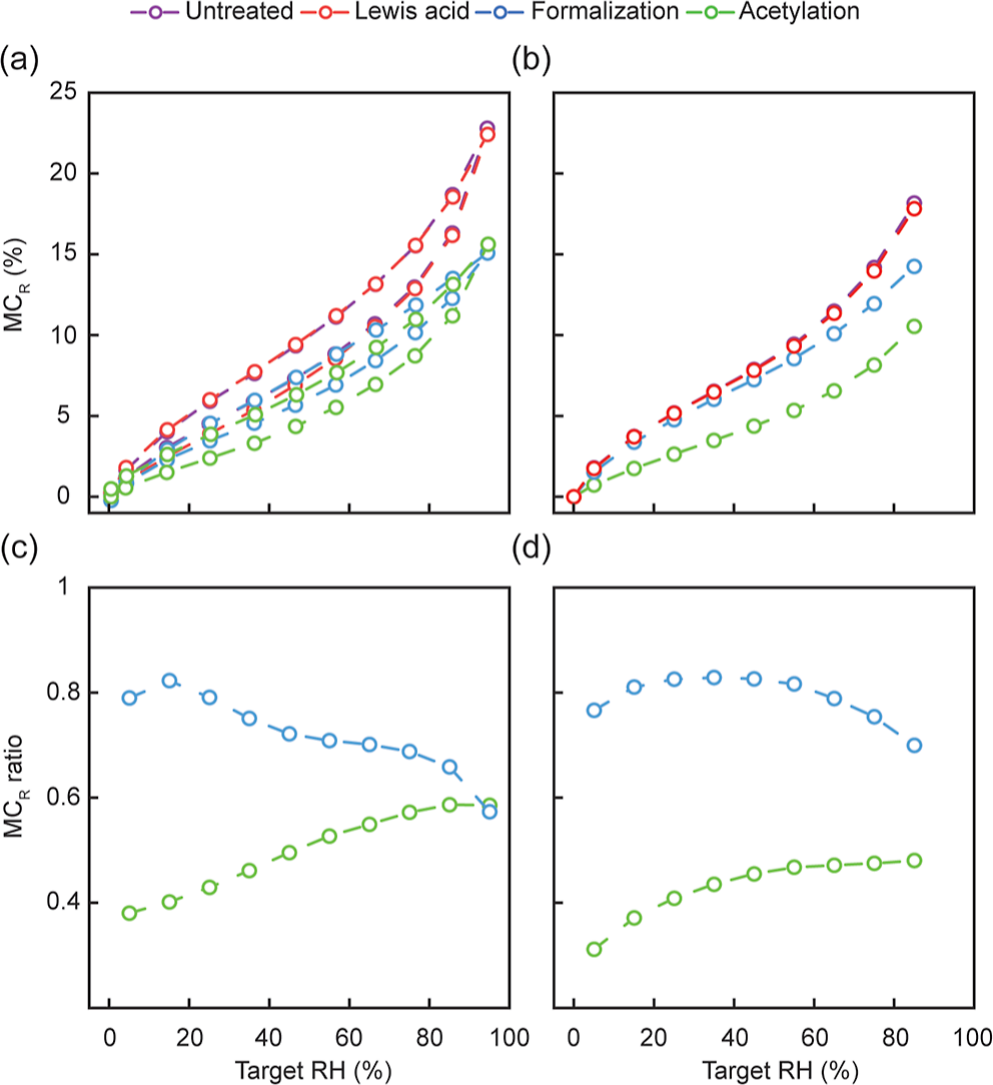
Sorption isotherms and moisture content ratios (MC ratios) measured using two different sorption balances. (a) Moisture content (%) in absorption and desorption from 0 to 95% RH measured on wood powder. (b) Moisture content (%) in absorption and desorption from 0 to 85% RH measured on larger wood blocks. (c) MC ratio of modified wood samples relative to untreated and Lewis acid-treated wood measured on wood powder. (d) MC ratio of modified wood samples relative to untreated and Lewis acid-treated wood measured on wood blocks.

The moisture content data shown in Figure 4 were corrected for the weight added by the treatments using the factor given by Eq 4. The absorption and desorption isotherms of untreated and Lewis acid-treated wood demonstrated typical sigmoidal shapes commonly observed in most cellulose-based materials^33^ (Figure 4a). At 65% RH, the upward bend in the isotherm of the untreated sample may be attributed to the softening of amorphous polymers such as hemicellulose in the cell wall.^20^ The softening decreases the overall viscosity and rigidity of the polymer matrix and creates additional space for the water molecules.^34^ There was a small difference in isotherms of the untreated reference and the Lewis acid-treated samples, which could be caused by acid-catalyzed hydrolysis of carbohydrate polymers of the cell wall.^21^ Acetylation and formalization reduced the wood moisture content over the entire hygroscopic range. Similar absorption isotherms were obtained from another sorption balance (SPSx-1μ-High-Load, Figure 4b). A comparison of absolute wood moisture contents between the two-sorption balance is, however, not meaningful because different sets of samples were measured.

Differences in the effectiveness of moisture content reduction at different RH levels were studied in more detail by use of the MC ratio, which relates the moisture content of the treated wood to the moisture content of the corresponding reference measured in absorption (Figure 4c,d). The MC ratio of acetylated wood increased with RH, but constant MC ratios were approached above 80% (Figure 4c) or 50% RH (Figure 4d), depending on which sorption balance was used. In contrast, the MC ratio of formalized wood increased slightly until 15–25% RH and then decreased continuously upon a further increase in RH. The magnitudes of the MC ratios differed between the two different sorption measurements, primarily because the samples differed in size, form, mass, and treatment level. However, the trends of increasing MC ratios for acetylated wood and decreasing MC ratios above 25% RH for formalized wood were observed for both measurements. Similar trends were reported by Himmel and Mai^20^ and were also obtained after calculation of MC ratios from the MC data published by Yasuda et al.^35^ These different trends in the modified samples are in line with the suggested mechanisms in acetylated and formalized wood. Acetylation reduces the concentration of accessible OH groups and the water-available cell wall space but does not restrict the swelling of the wood cell wall. Swelling in the amorphous cell wall increases the free-volume and chain mobility, thus facilitating moisture uptake at elevated RH.^36^ The presumed cross-linking between the cell wall polymers in formalized wood restricts swelling. The increased stiffness of the cross-linked cell wall matrix reduces water incorporation, and this effect becomes more dominant with increasing RH.^20^

### 3.5 Simultaneous Mass and Dimensional Changes

**Figure 5:**
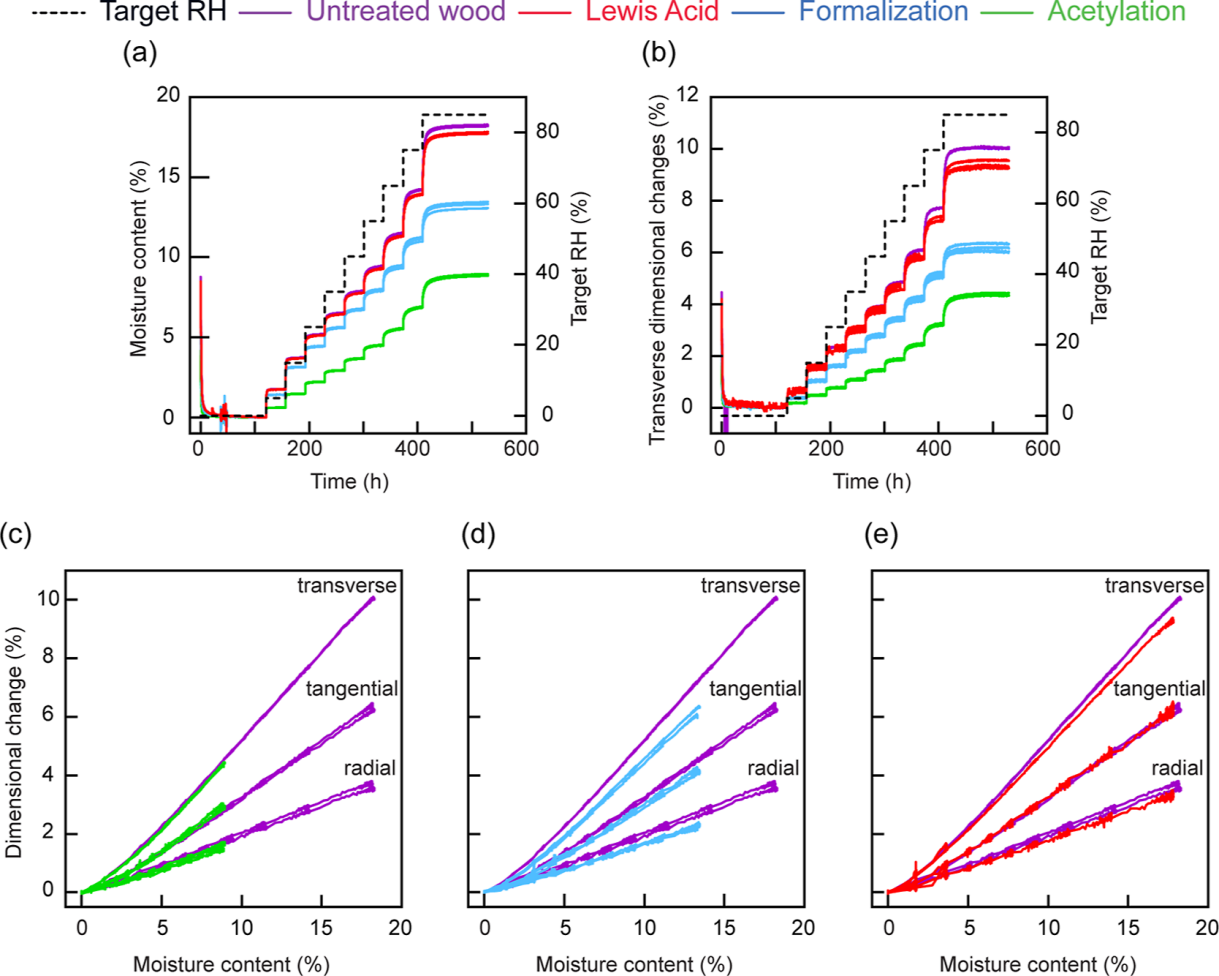
Simultaneous moisture content and dimensional changes during exposure to 0–85% RH. Changes in moisture content (%) and target RH (%) over time (hours) (a). Changes in transverse swelling (%) and target RH (%) over time (hours) (b). Swelling (transverse, tangential, and radial) as a function of moisture content during absorption in untreated wood and acetylated (c), formalized (d), or Lewis-acid treated (e) wood.

Water vapor sorption and the consequent dimensional changes are shown in Figure 5. The course of moisture content (Figure 5a) and dimensional changes (Figure 5b) over time were similar. Both showed a fast increase from one RH step to the next and approached constant values when a stable RH was held. Moisture content and dimensional changes were reduced by the treatments, with the Lewis acid treatment resulting in the smallest reduction and acetylation in the strongest reduction. It should be noted, however, that the moisture content and dimensional changes shown in Figure 5 were not corrected for the increase in dry mass and dry dimensions caused by the treatments. A comparison of the absolute moisture content and dimensional changes is thus not meaningful due to the differences in WPG and cell wall bulking of the different treatments.

Instead, the treatments were compared by relating dimensional changes to the corresponding moisture content at each time point (Figure 5c–e). There was a strong linear correlation between the dimensional changes and the moisture content over the MC range of 5–20%, showing that each water molecule caused swelling in the untreated wood.^23^ At lower moisture content (0–5%), the absorption behavior was slightly non-linear. The anisotropic behavior of wood also resulted in a stronger increase in dimensions in the tangential direction compared to the radial direction.^6^ Acetylation did not change the slope at which the dimensions increased as a function of moisture content compared to untreated wood (Figure 5c). The existence of acetyl groups within the cell walls increased the dry dimensions, which reduced the volumetric margin between the dry and the wet state without restricting the swelling of the cell wall. Each absorbed water molecule in acetylated wood caused the same increase in dimensions as recorded for untreated wood.

In contrast, the reduction of the dimensional changes in formalized wood was not proportional to the reduction in moisture content (Figure 5d). The slope of dimensional changes as a function of moisture content was reduced compared to untreated wood, and this was observed in both radial and tangential directions. A small reduction in slope was also observed in Lewis acid-treated wood. The acid-catalyzed hydrolysis of cell wall components may thus have contributed to the behavior of formalized wood. It is also possible that the formaldehyde- and Lewis acid-treated samples showed out-of-plane deformation during the sorption measurements, which was not recorded from the top view of the camera. However, the reduced slope may also be related to the formation of additional cross-links in the cell wall matrix of formalized wood that restrict swelling. Moisture content-dependent changes in wood dimensions also differ between wood species and are also influenced by the state of sorption.^37,38^ The swelling of wood is a complex phenomenon that is closely linked to the multilayered cell wall structure and the stiffness circumferential and perpendicular to the cell wall.^39^ The formalization of wood may affect this and could be a tool to better understand the influence of the (multilayered) cell wall structure on the wood–water relationship.

## 4 Conclusions

Wood samples were modified by acetylation and formalization to change their sorption behavior. Both modifications reduced water uptake by the wood material, but notable differences could be seen in their sorption isotherms and swelling behavior. Acetylation reduced moisture content more strongly at low rather than high relative humidity and did not affect the swelling of wood as a function of moisture content, while formalization reduced the moisture content more strongly at high rather than low relative humidity and reduced swelling as a function of moisture content. These findings support the understanding that acetylation reduces water uptake primarily by cell wall bulking without swelling restriction, while formalization also provides additional cross-linking that restricts swelling. The results presented here demonstrate that moisture uptake and swelling in wood are complex phenomena that are strongly dependent on the structure and composition of the wood cell wall. Wood modification appears to be a promising tool to alter the cell wall structure to better understand wood–water interactions.

## Supporting Information Available

Camera images equipped with automated sorption balance reference sample image transformed into outline (PNG) and sample outline before (dry state) and after (wet state) the swelling, indicating dimensional changes (PDF)

## Notes

Numerical computation and illustrations: Numerical computation and data plotting were performed on commercial software packages MATLAB 2020b (The MathWorks, Inc.) and OriginPro (OriginLab Corp.). The vector illustrations were prepared using the software Adobe Illustrator.

## Notes

Data availability: Sorption data and in-house scripts can be provided for research purposes by the corresponding author on reasonable request.
