# Supplementary material for: Wood–Water Relations Affected by Anhydride and Formaldehyde Modification of Wood
Source: ACS Omega. 2022 Nov 8;7(46):42199–207. doi: 10.1021/acsomega.2c04974 (PMC9685604; doi:10.1021/acsomega.2c04974)
Supplement: Supplementary file 1 — ao2c04974_si_001.pdf [file ao2c04974_si_001.pdf]

Supporting Information

Wood-water relations affected by anhydride and formaldehyde modification of wood

Muhammad Awais[*a], *Michael Altgen*[a,b], *Tiina Belt*[a,c], *Venla Teräväinen*[a], *Mikko Mäkelä*[d], *Daniela Altgen*[a], *Martin Nopens*[e], *Lauri Rautkari*[a]

[a] *Aalto University, Department of Bioproducts and Biosystems, PO Box 16300, 00076, Aalto, Finland*

[b] *Universität Hamburg, Department of Biology, Institute of Wood Science, Leuschnerstraße 91c, 21031 Hamburg, Germany*

[c] *Natural Resources Institute Finland (Luke), Viikinkaari 9, 00790 Helsinki, Finland*

[d] *VTT Technical Research Centre of Finland Ltd, P.O. Box 1000, FI-02044, VTT, Finland*

[e] *Johann Heinrich Von Thünen Institute, Federal Research Institute for Rural Areas, Forestry and Fisheries, Institute of Wood Research, Leuschnerstrasse 91, 21031 Hamburg, Germany*

Corresponding Author

* Tel.: +358413698110. E-mail: Muhammad.Awais@aalto.fi

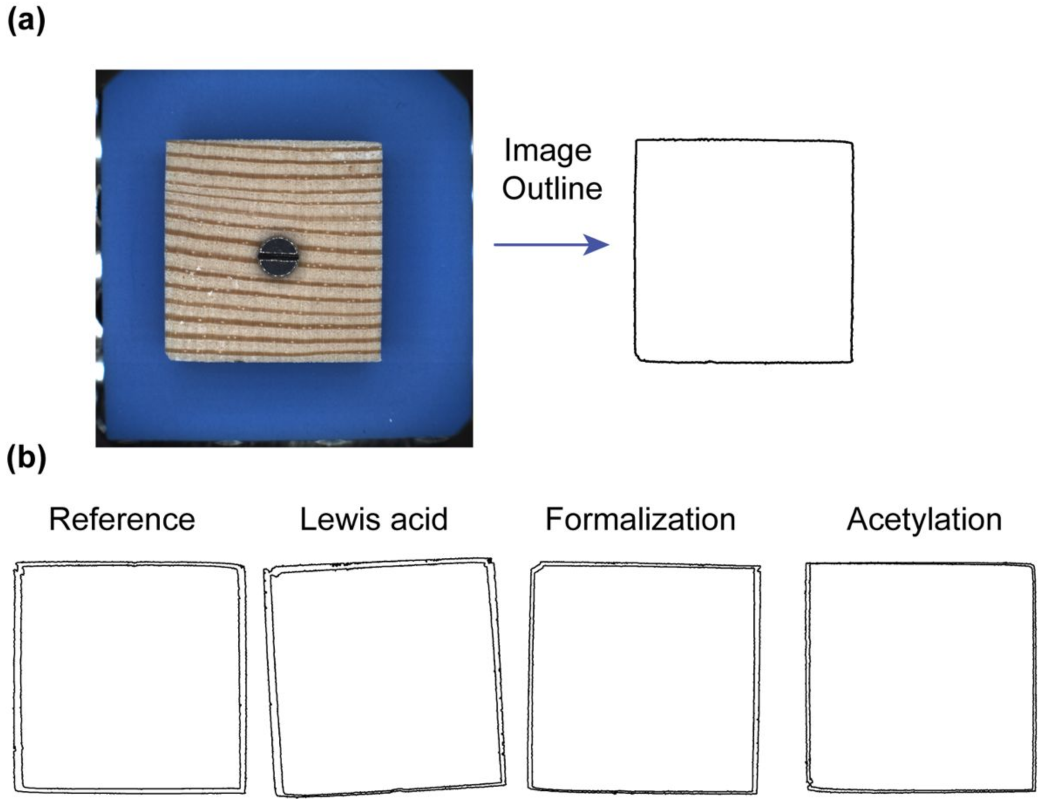

Figure S1. Camera images equipped with automated sorption balance (a) Reference sample image transformed into outline (PNG) (b) Samples outline before (dry state) and after (wet state) the swelling indicating dimensional changes.
